# Supplementary material for: Deciphering the antigen specificities of antibodies by clustering their complementarity determining region sequences
Source: mSystems. 2023 Nov 17;8(6):e00722-23. doi: 10.1128/msystems.00722-23 (PMC10734444; doi:10.1128/msystems.00722-23)
Supplement: Fig. S1 to S3 and Tables S1 to S3 — Additional experimental details and statistical analysis. [file msystems.00722-23-s0001.docx]

**Supplemental Figures**

**
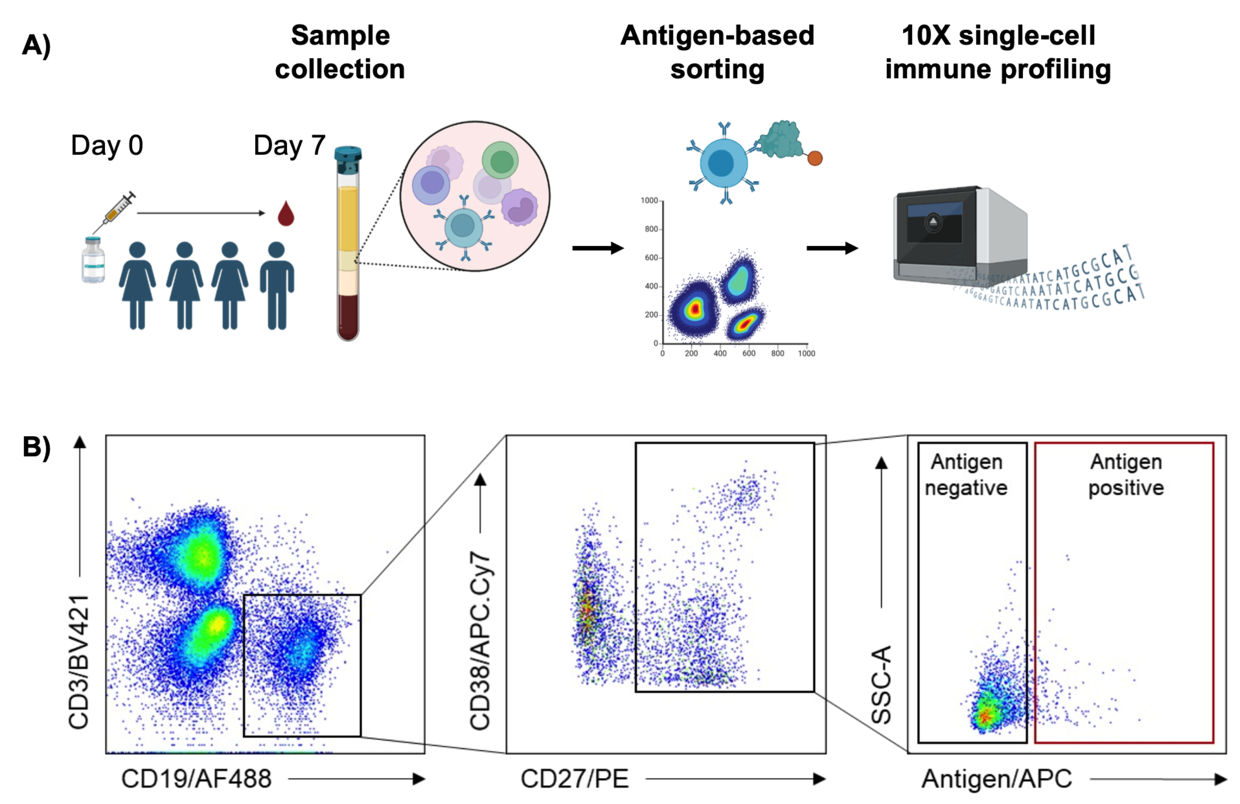
**

**FIG S1. Profiling immune responses to DTP vaccination.** (A) Sample collection. Four healthy adult donors were recruited and vaccinated by DTP booster (TRIBIK, BIKEN). Isolation of serum and PBMCs was done on 7 days post vaccination. PBMCs were sorted by fluorescently-labeled toxins to get DTP binding and not binding B cell and the sample were sent for 10x Genomics single-cell sequencing. (B) Gating strategy to sort DTP toxin-binding and non-binding antigen-experienced B cells from post-vaccine PBMCs. First, the population of B cells (CD19^+^CD3^-^) was gated from total PBMCs containing mixture of four donors. Then, the CD27^+^ cells including the CD38^hi/lo^ was gated to obtain antigen experienced B cells and followed by double negative controls gating to sort the toxin-binding (DT, TT, or PT) and non-binding (NB) cells.


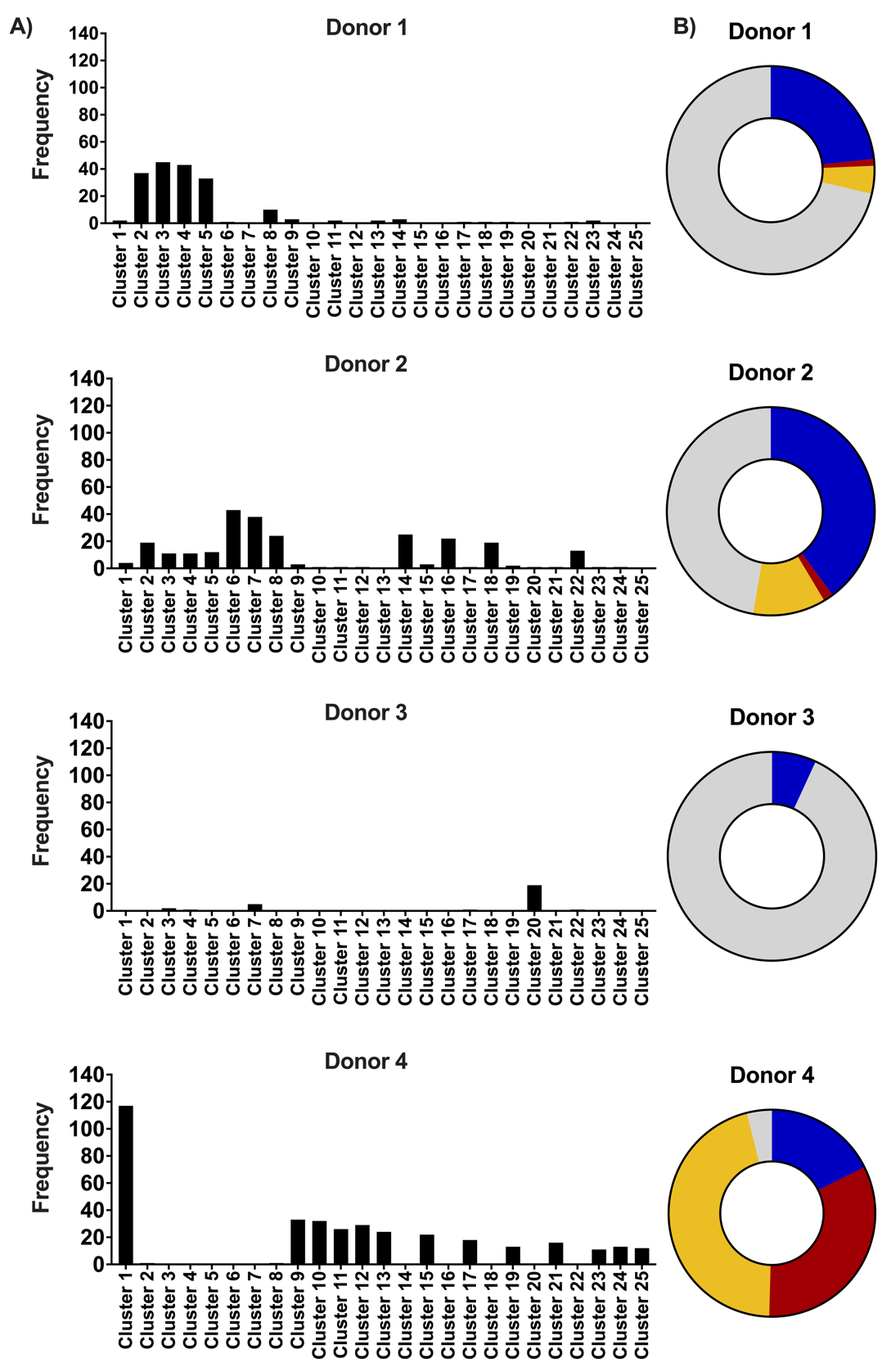


**FIG S2. Dominant cluster analysis across donors.** A) Different levels of repertoire responses among multiple donors showed as histograms of sequence distribution in Clusters 1-25. B) After de-orphaning the dominant clusters, we plot the distribution of antigen-specific response in Cluster 1-25 among donors. Each donor showed a distinct pattern of antigen-specific response to the same vaccine, showed in the pie charts. The number of n for Donor 1, 2, 3, and 4 are 185, 256, 29, and 357, respectively.


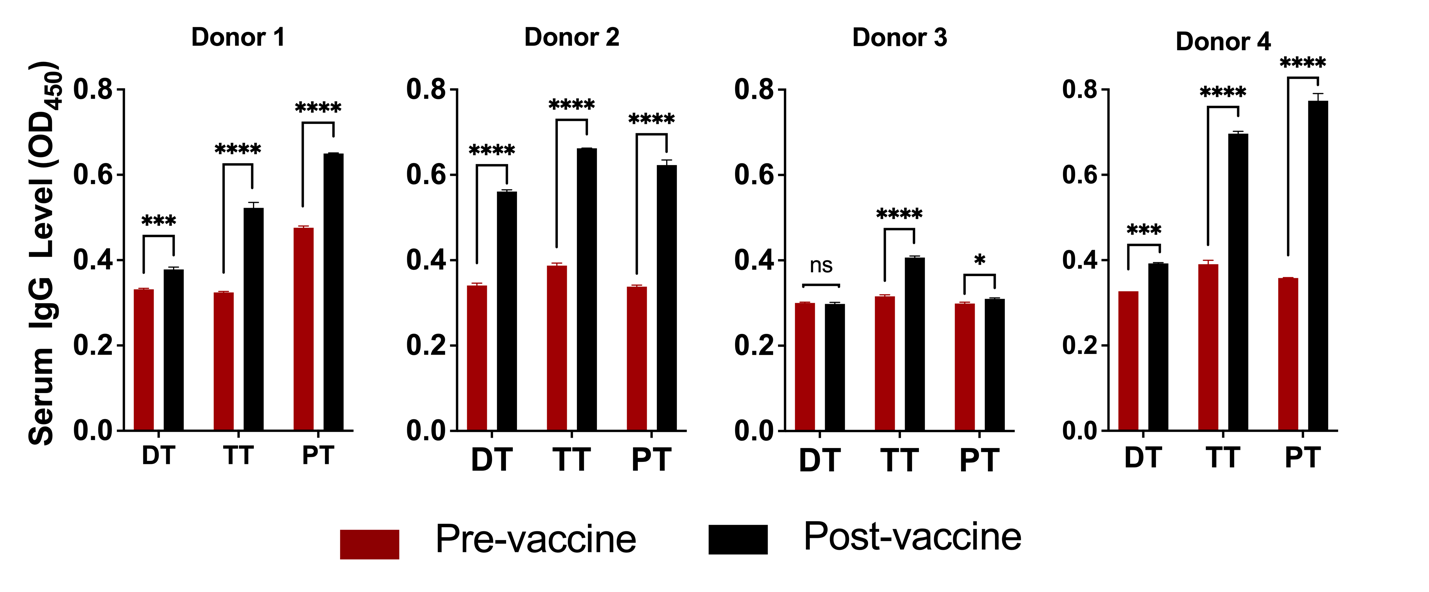


**FIG S3.** **Humoral immune response among donors, analyzed by ELISA.** Values represent mean ± SD of OD_450_ in duplicated wells. Statistical differences in were analyzed by two-way analysis of variance with Šídák’s multiple-comparison test: *, P < 0.01; **, P < 0.001; ***, P < 0.0001; ns, not significant.

**Supplemental tables**

**Table S1. Cluster purity score resulted from different sequence identity and coverage thresholds**

| **SID**  **COV (%)**  **(%)** | 70 | 75 | **80** | 85 | 90 | 95 | 100 |
| --- | --- | --- | --- | --- | --- | --- | --- |
| 100 | 0.950 | 0.961 | 0.968 | 0.975 | 0.978 | 0.986 | 0.982 |
| 95 | 0.920 | 0.946 | 0.96 | 0.973 | 0.978 | 0.985 | 0.983 |
| **90** | 0.898 | 0.934 | **0.953** | 0.971 | 0.979 | 0.985 | 0.983 |
| 85 | 0.885 | 0.930 | 0.949 | 0.971 | 0.978 | 0.986 | 0.983 |
| 80 | 0.875 | 0.915 | 0.940 | 0.964 | 0.974 | 0.983 | 0.983 |
| 75 | 0.854 | 0.893 | 0.917 | 0.950 | 0.968 | 0.979 | 0.979 |
| 70 | 0.827 | 0.873 | 0.896 | 0.922 | 0.946 | 0.964 | 0.969 |

Chosen sequence identity (SID) and coverage (COV) threshold is indicated in bold with resulting cluster purity in grey shaded cell.

**Table S2. Clonotyping vs CDR clustering contingency table**

|  | **Non-singleton group** | **Singleton group** | **Total** |
| --- | --- | --- | --- |
| **Clonotype** | 952 | 10,684 | 11,636 |
| **CDR cluster** | 1,198 | 9,604 | 10,802 |
|  |  |  | 22,438 |

Chi-square test results: x^2^ = 54.72; df = 1; p <0.0001

**Table S3. Clonotyping vs CDR clustering contingency table**

|  | **Public** | **Private** | **Total** |
| --- | --- | --- | --- |
| **Clonotype** | 270 | 11,366 | 11,636 |
| **CDR cluster** | 392 | 10,410 | 10,802 |
|  |  |  | 22,438 |

Chi-square test results: x^2^ = 33.50 df = 1; p <0.0001
